# Meta-analysis derived atopic dermatitis (MADAD) transcriptome defines a robust AD signature highlighting the involvement of atherosclerosis and lipid metabolism pathways

**DOI:** 10.1186/s12920-015-0133-x

**Published:** 2015-10-12

**Authors:** David A. Ewald, Dana Malajian, James G. Krueger, Christopher T. Workman, Tianjiao Wang, Suyan Tian, Thomas Litman, Emma Guttman-Yassky, Mayte Suárez-Fariñas

**Affiliations:** The Laboratory for Investigative Dermatology, The Rockefeller University, New York, NY USA; Department of Dermatology, Icahn School of Medicine at Mount Sinai, New York, NY USA; Molecular Biomedicine, LEO Pharma AS, Ballerup, Denmark; Center for Biological Sequence Analysis, Department of Systems Biology, Technical University of Denmark, Kgs. Lyngby, Denmark; Columbia University, College of Physicians and Surgeons, New York, NY USA; School of Life Science, Jilin University, 2699 Qianjin Street, Changchun, Jilin 130012 China; Department of Population Health Science and Policy, Icahn School of Medicine at Mount Sinai, New York, NY USA; Icahn Institute for Genomics and Multiscale Biology at Mount Sinai, Icahn School of Medicine at Mount Sinai, New York, NY USA

**Keywords:** Atopic dermatitis, Meta-analysis, Transcriptome, Atherosclerosis, Expression analysis

## Abstract

**Background:**

Atopic dermatitis (AD) is a common inflammatory skin disease with limited treatment options. Several microarray experiments have been conducted on lesional/LS and non-lesional/NL AD skin to develop a genomic disease phenotype. Although these experiments have shed light on disease pathology, inter-study comparisons reveal large differences in resulting sets of differentially expressed genes (DEGs), limiting the utility of direct comparisons across studies.

**Methods:**

We carried out a meta-analysis combining 4 published AD datasets to define a robust disease profile, termed meta-analysis derived AD (MADAD) transcriptome.

**Results:**

This transcriptome enriches key AD pathways more than the individual studies, and associates AD with novel pathways, such as atherosclerosis signaling (IL-37, selectin E/SELE). We identified wide lipid abnormalities and, for the first time in vivo, correlated Th2 immune activation with downregulation of key epidermal lipids (FA2H, FAR2, ELOVL3), emphasizing the role of cytokines on the barrier disruption in AD. Key AD “classifier genes” discriminate lesional from nonlesional skin, and may evaluate therapeutic responses.

**Conclusions:**

Our meta-analysis provides novel and powerful insights into AD disease pathology, and reinforces the concept of AD as a systemic disease.

**Electronic supplementary material:**

The online version of this article (doi:10.1186/s12920-015-0133-x) contains supplementary material, which is available to authorized users.

## Background

Atopic dermatitis (AD) is the most common inflammatory skin disease (4–7 % prevalence in adults, and ~15 % in children), with a large unmet need for safer and more effective treatments [1–7]. Immune and barrier abnormalities characterize AD, with Th2/Th22 cytokine activation, increased hyperplasia and significant decreases in differentiation markers. These observations have led to two competing pathogenic hypotheses [1, 8, 9], although recent studies characterizing AD primarily as an immune-driven disease have shown reversal of barrier defects following specific and non-specific therapeutic interventions [10–13].

Genomic expression profiling using gene-arrays and real time (RT)-PCR has been widely used to identify gene alterations in lesional (LS) and non-lesional (NL) AD compared to normal skin to better understand interactions between activation of cytokine pathways and epidermal abnormalities [6, 7, 10, 12, 14, 15]. Similar to other diseases, the AD phenotype/transcriptome can be defined genomically by differentially expressed genes (DEGs) between LS and NL skin [15]. A robust transcriptome was established as a powerful tool in identifying core psoriasis pathogenesis and evaluating the efficacy of targeted therapeutics at transcriptomic level [16, 17]. The high rates of placebo effect in AD patients contrasts with a worsening of disease phenotype at the transcriptomic level [10], reinforcing the importance of a robust disease transcriptome against which therapeutic effects can be evaluated [18]. Genomic profiling may also be used to predict therapeutic responses, as in juvenile idiopathic arthritis, in which profiling correctly predicted therapeutic responses at 6 months [19].

Nevertheless, high-throughput genomic analyses are vulnerable to multiple biases, including random noise, biological heterogeneity, and differences in experimental procedures (biopsies, hybridization, etc.), leading to remarkably little overlap between DEGs in similar scale studies [20–22]. A meta-analysis approach that combines microarray data from independent yet similarly designed studies allows one to overcome these variations, ultimately increasing the power and reproducibility of the transcriptome [23–25]. While several meta-analysis methods exist for combining microarray data from independent studies [26], the random-effects model (REM) has been established as one of the most suitable for heterogeneous studies [25, 27, 28].

We applied a REM meta-analysis model including 4 published AD microarray studies (including 97 samples; 54 LS and 43 NL; 41 paired) to determine core pathogenic elements and new disease associated genes [9, 10, 12, 15], resulting in the Meta-Analysis Derived AD (MADAD) disease transcriptome, a robust active disease signature of 595 DEGs, including 86 that were not previously detected by any individual study.

## Methods

### Sample collection

All samples were collected according to the Preferred Reporting Items for Systematic Reviews and Meta-Analyses (PRISMA) statement [29]. A total of 28 datasets were detected in Gene Expression Omnibus (GEO), but only datasets including LS and NL skin samples of AD patients were retained. Datasets run on platforms other than the HGU133Plus 2 chip, subject to treatments, or with non-randomly selected NL or LS samples (e.g. FLG homozygous/heterozygous loss of function mutation), and datasets without NL samples were excluded. When overlapping samples were found between datasets, only one copy was kept. Four microarray datasets satisfied the established criteria (GSE32924, GSE36842, GSE58558, GSE59294) [9, 10, 12, 15], including 97 samples (54 LS and 43 NL), which coincidently have been carried out by our group.

### Pre-processing and expression analysis

Pre-processing and statistical analyses were performed using *R* and *Bioconductor* packages [30, 31]. Raw expression data were combined, summarized, and normalized using *GCRMA* [32]. Batch effects between datasets were adjusted for by the empirical Bayes method *ComBat/SVA* [32–35]. Agreement of the individual studies raw microarray data was estimated by the Integrated Correlation Coefficient Analysis, which produces the general Integrated Correlation Coefficient (ICC), representing agreement between studies, and can be interpreted in the same way as Pearson correlation coefficient. The ICC was used to eliminate background noise prior to the analysis, by excluding genes with incoherent behavior across studies [36].

For each study, estimation of differences in expression levels of LS vs NL skin was performed using the mixed-effect framework of the *limma* package.

### Meta-analysis

The formal *random effects model (REM)*, described by Choi [27]. was applied to estimate the true effect size for each probe (see Additional file 1). These estimation and calculation steps were performed using the package *GeneMeta.* P-values were adjusted for multiple testing using the *Benjamini-Hochberg procedure* [37], with criteria for DEGs of absolute fold change (|FCH|) ≥ 2.0 and a false discovery rate (FDR) ≤ 0.05.

### Post-processing

The MADAD transcriptome was subject to multiple downstream analysis methods. Integration-driven discovery/IDD-DEGs were defined as those not identified in any of the included studies. To explore functional annotations, overrepresentation analysis was carried out for BP GO-terms and KEGG pathways (both in DAVID) [38], Ingenuity Pathways (*IPA –*www.ingenuity.com, as described) [25], and on previously reported gene-sets [39].

The normalized LS and NL expression data were subject to Weighted Gene Co-Expression Networks Analysis (WGCNA) to detect clusters (modules) of correlated genes and respective hub genes [40]. These modules were subject to trait correlation and corresponding gene-set overrepresentation analysis.

Meta Threshold Gradient Directed Regularization (MTGDR) method [41, 42] was used to select disease-associated genes while allowing for varied estimates of those genes across different experiments, as previously published [41, 42].

Primers and probes were designed for RT-PCR as previously described (see Additional file 1 and Additional file 2) [43]. *Ribosomal protein, large, P0*/RPLP0 normalized RT-PCR expression data was analyzed using a mixed-effect framework after log2-transformation.

Lipid metabolism genes were defined as genes related to one of the four groups: *Ceramides*, *Free Fatty Acids*, *Sphingolipids*, and *Cholesteryl Esters* in the Gene Cards database (www.genecards.org). We included all genes with a relevance score ≥ 10 [44, 45]. Pairwise Pearson correlations were calculated between the gene and patient specific deregulations. Multivariate correlations between sets of genes were calculated making use of the gene set specific μ-scores calculated by the *muStat* package [46].

## Results

### Data collection

Two major repositories (GEO Omnibus and ArrayExpress) were queried to identify studies with expression profiles of LS and NL punch biopsies from AD patients. Four studies (GSE59294, GSE58558, GSE36842, GSE32924; including 97 samples, with 54 LS and 43 NL, 41 paired) met all inclusion criteria following the PRISMA guidelines (see Methods, Additional file 3: Figure E1 and Additional file 4: Table E1) [9, 10, 12, 15]. We only used data from chronic lesions in the analysis. No significant differences in disease severity (as measured by Scoring of AD/SCORAD and Eczema Area and Severity/EASI indices) or IgE levels were found across patients who all had moderate-to-severe AD (SCORAD > 25; EASI > 12).

### Meta-analysis framework

**Fig. 1:**
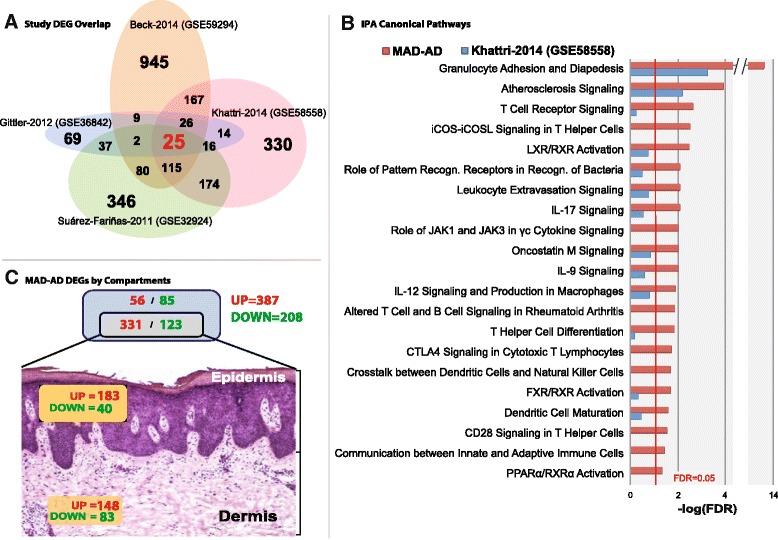
**a** Approximate area proportional Venn diagram with overlaps of the differentially expressed genes of the four individually analyzed datasets under the same threshold (|FCH| ≥ 2, FDR ≤ 0.05). **b** Ingenuity Canonical Pathway overrepresentations compared between this MADAD transcriptome and the included dataset from Khattri et al. 2014 (GSE58558). The bars represent –log10 transformed Benjamini-Hochberg adjusted p-values termed –log(FDR), and the red threshold line indicates the FDR ≤ 0.05 cut-off. **c** Cutaneous localization of the MADAD transcriptome as compared to the epidermis and dermis related genes defined by Esaki et al. [63]

The Venn diagram in Fig. 1a represents the overlap of DEGs identified by the individual studies, including 25 consensus DEGs (see Additional file 5: Table E2). Besides inter-study variation, sources of inconsistency in DEGs include choice of model, annotation, cut-off, and non-uniform pre-processing steps [47]. To combine results of individual studies and to aggregate robust DEGs with reliable effect sizes, we chose a meta-analysis approach [21, 27].

To address these sources of inconsistency, we applied a uniform pre-processing pipeline to the combined datasets, incorporating noise reduction techniques as proposed [23, 25, 36, 47] (see Methods, Additional file 3: Figure E1C, and Additional file 1). This pre-processing increased the average of the pairwise maximum canonical correlations between each pair of studies from 0.76 to 0.85.

To combine heterogeneous effect sizes across studies, a random effects model/REM was chosen over fixed effects model. The use of REM is supported by the sample distribution of the Cochran’s Q statistic [27], which departs substantially from the theoretical *χ*_3_^2^-distribution under the assumption of homogeneity of study effects. Furthermore, a Kolmogorov-Smirnov-test also rejected the equality of these two distributions (*p* = 2.22^−15^; D = 0.235) (Additional file 6: Figure E2).

### The Meta-analysis-derived AD (MADAD) transcriptome

Applying the meta-analysis approach to 4 studies and 97 samples, we identified a set of 595 DEGs (387 up- and 208 downregulated) using the classical |FCH| ≥ 2 and FDR ≤ 0.05 criteria, representing a robust profile defined as the meta-analysis derived AD (MADAD) transcriptome (see Additional file 7: Table E3 for the entire MADAD DEGs list). Among the highest dysregulated genes are key AD genes, including markers of general inflammation (MMP12), specific T helper activation (e.g. Th2/CCL18, Th1/IFN/CXCL10, Th17/PI3/elafin, Th17/Th22 S100A7/A8/A9), and markers of epidermal proliferation (KRT16, Mki67).

To show the robustness of the MADAD transcriptome versus a single study, we conducted an over-representation analysis (see Methods) of ingenuity canonical pathways (IPA) and of previously published immune and barrier gene-sets [12, 48]. Figure 1b illustrates the comparison of the largest available’ dataset (Khattri 2014 – GSE58558) and the MADAD transcriptome; similar results were obtained for the other three included datasets. Overall, the MADAD yields a more significant over-representation of key immune pathways, such as *Granulocyte Adhesion and Diapedesis, T Cell Receptor Signaling and differentiation*, *iCOS-iCOSL Signaling*, and *IL-12, IL-17,* and *IL-9 signaling*. Innate pathways (e.g. *Role of Pattern Recognition Receptors in Recognition of Bacteria*), which are associated with AD [49, 50], were also represented (Fig. 1b and Additional file 8: Tables E4 and Additional file 9: Table E5). Interestingly, *Atherosclerosis Signaling* was the second highest IPA pathway, and includes genes previously associated with vascular inflammation, such as IL-37, SERPINA1, S100A8, selectin E/SELE, lipoprotein lipase/LPL, and MMP1/3/9 [51–62]. An equivalent analysis of previously reported immune and barrier gene-sets [12] (Additional file 10: Figure E3A) similarly showed increased sensitivity and representation in the MADAD transcriptome compared to the largest data set, including IFNα, IL-4, immune genes, cytokine-treated keratinocytes and epidermal differentiation gene subsets (Additional file 10: Figure E3A). Thus, the MADAD transcriptome provides a more robust AD-specific signal, consistent with known disease pathology.

Comparison of the MADAD transcriptome to the recently described epidermal and dermal layer-specific transcriptomes linked 223 and 231 DEGs to the epidermis and dermis, respectively (Fig. 1c, Additional file 7: Table E3) [63]. Of these, 55.3 % of the upregulated genes were epidermal, whereas 67.5 % of the downregulated genes were dermal (*P* = 2.6 × 10^−5^, Fisher’s exact test), as has been previously noted [63].

Of the top 25 up-regulated MADAD DEGs, 17 encode epidermal components, including key antimicrobial genes (DEFB4A, PI3/elafin, S100A9) (Additional file 7: Table E3). Top 25 up-regulated dermal genes include those related to collagen production (COL4A4, COL6A5) and inflammation (GZMB, OASL) [64, 65]. Among top down-regulated epidermal genes are structural/lipid-related genes (LEP, FABP7, ELOVL3), and genes linked to epidermal differentiation (CLDN8) [66–68]. Several genes associated with the pathogenesis of AD (e.g. IL-22, OX40L and TSLP) and reported in the layer-specific AD transcriptomes [63] were not detected in the MADAD transcriptome, most likely due to low expression levels of cytokines on whole tissue microarrays, a known limitation of microarrays [24, 69], that cannot be overcome by the meta-analysis approach.

### Integration-Driven Discovery Genes in the MADAD transcriptome

The MADAD comprises a subset of IDD-DEGs, which were not detected by any of the individual datasets using identical cutoffs [9, 10, 12, 15]. These 86 IDD-DEGs consist of 45 up- and 41 downregulated genes (Table 1). Using IPA and GOterm overrepresentation analyses on the IDD-DEGs, we found in addition to the expected inflammatory processes (e.g. Chemokine Signaling), several pathways related to lipid and fatty acid metabolic processes (Additional file 10: Figure E3B, Additional file 11: Table E6).

**Table 1:** Integration-Driven Discovery (IDD) Genes in the MADAD transcriptome, with indication of compartmental allocation as defined by Esaki et al. [63]

Symbol	Description	logFC	FC	Layer
Up				
COL6A6	collagen, type VI, alpha 6	2,08	4,22	D
CD1B	CD1b molecule	1,84	3,57	D
SPRR1B	small proline-rich protein 1B	1,68	3,20	E
CCL22	chemokine (C-C motif) ligand 22	1,65	3,13	D
MMP9	matrix metallopeptidase 9 (gelatinase B, 92 kDa gelatinase, 92 kDa type IV collagenase)	1,51	2,84	D
IL13RA2	interleukin 13 receptor, alpha 2	1,46	2,74	D
CCL26	chemokine (C-C motif) ligand 26	1,37	2,59	D
SASH3	SAM and SH3 domain containing 3	1,35	2,56	D
IL36RN	interleukin 36 receptor antagonist	1,35	2,55	E
CCL13	chemokine (C-C motif) ligand 13	1,27	2,41	D
KIAA1644	KIAA1644	1,26	2,39	D
IL12RB1	interleukin 12 receptor, beta 1	1,23	2,34	D
XCL2	chemokine (C motif) ligand 2	1,21	2,31	D
CCL5	chemokine (C-C motif) ligand 5	1,21	2,31	D
ADAMDEC1	ADAM-like, decysin 1	1,19	2,29	D
TIFAB	TRAF-interacting protein with forkhead-associated domain, family member B	1,18	2,27	D
P2RY1	purinergic receptor P2Y, G-protein coupled, 1	1,16	2,23	E
PIK3CG	phosphatidylinositol-4,5-bisphosphate 3-kinase, catalytic subunit gamma	1,15	2,22	D
FAM124B	family with sequence similarity 124B	1,14	2,21	D
SLAMF8	SLAM family member 8	1,12	2,18	D
CXADR	coxsackie virus and adenovirus receptor	1,12	2,17	E
GPSM3	G-protein signaling modulator 3	1,11	2,16	D
HCK	hemopoietic cell kinase	1,09	2,13	D
LOC100288860	uncharacterized LOC100288860	1,09	2,12	E
MMP3	matrix metallopeptidase 3 (stromelysin 1, progelatinase)	1,08	2,11	D
CD1E	CD1e molecule	1,07	2,10	D
KLRK1	killer cell lectin-like receptor subfamily K, member 1	1,06	2,09	D
GBP1	guanylate binding protein 1, interferon-inducible	1,06	2,09	
IL23A	interleukin 23, alpha subunit p19	1,05	2,08	D
LYN	v-yes-1 Yamaguchi sarcoma viral related oncogene homolog	1,05	2,07	D
C5orf20	chromosome 5 open reading frame 20	1,05	2,06	D
CCL8	chemokine (C-C motif) ligand 8	1,04	2,06	D
RELB	v-rel avian reticuloendotheliosis viral oncogene homolog B	1,04	2,06	
ACPP	acid phosphatase, prostate	1,03	2,05	E
TRAT1	T cell receptor associated transmembrane adaptor 1	1,03	2,04	D
PTX3	pentraxin 3, long	1,03	2,04	D
CD48	CD48 molecule	1,03	2,04	D
FPR3	formyl peptide receptor 3	1,02	2,03	D
TGM3	transglutaminase 3	1,02	2,03	E
CXCL11	chemokine (C-X-C motif) ligand 11	1,02	2,03	
MAP4K1	mitogen-activated protein kinase kinase kinase kinase 1	1,02	2,02	D
CD6	CD6 molecule	1,02	2,02	D
SELPLG	selectin P ligand	1,01	2,02	D
ZC3H12D	zinc finger CCCH-type containing 12D	1,01	2,01	D
C15orf48	chromosome 15 open reading frame 48	1,00	2,00	E
Down				
PM20D1	peptidase M20 domain containing 1	−2,63	−6,19	
KRT79	keratin 79	−2,04	−4,13	
GAL	galanin/GMAP prepropeptide	−2,02	−4,06	
ELOVL3	ELOVL fatty acid elongase 3	−1,71	−3,26	
CYP4F8	cytochrome P450, family 4, subfamily F, polypeptide 8	−1,64	−3,11	
HAO2	hydroxyacid oxidase 2 (long chain)	−1,63	−3,10	
FADS2	fatty acid desaturase 2	−1,49	−2,80	
ANGPTL7	angiopoietin-like 7	−1,40	−2,63	D
CUX2	cut-like homeobox 2	−1,36	−2,56	
PON3	paraoxonase 3	−1,35	−2,55	E
SGK2	serum/glucocorticoid regulated kinase 2	−1,32	−2,50	
MSMB	microseminoprotein, beta-	−1,31	−2,47	E
FADS1	fatty acid desaturase 1	−1,30	−2,46	D
BPY2	basic charge, Y-linked, 2	−1,29	−2,45	
FAR2	fatty acyl CoA reductase 2	−1,29	−2,44	D
MUC7	mucin 7, secreted	−1,23	−2,35	
FA2H	fatty acid 2-hydroxylase	−1,20	−2,30	
ABHD12B	abhydrolase domain containing 12B	−1,18	−2,27	E
PNPLA3	patatin-like phospholipase domain containing 3	−1,18	−2,27	E
ACOX2	acyl-CoA oxidase 2, branched chain	−1,11	−2,16	D
PSORS1C2	psoriasis susceptibility 1 candidate 2	−1,10	−2,15	E
KRT19	keratin 19	−1,08	−2,11	
ATP6V1B1	ATPase, H+ transporting, lysosomal 56/58 kDa, V1 subunit B1	−1,07	−2,10	
SCGB2B2	secretoglobin, family 2B, member 2	−1,07	−2,09	
MOGAT1	monoacylglycerol O-acyltransferase 1	−1,07	−2,09	
NSUN7	NOP2/Sun domain family, member 7	−1,07	−2,09	E
COCH	cochlin	−1,05	−2,07	E
IL20RA	interleukin 20 receptor, alpha	−1,05	−2,07	E
SEMA3B	sema domain, immunoglobulin domain (Ig), short basic domain, secreted, (semaphorin) 3B	−1,03	−2,05	D
RHPN2	rhophilin, Rho GTPase binding protein 2	−1,03	−2,05	
AWAT1	acyl-CoA wax alcohol acyltransferase 1	−1,03	−2,04	
TMC4	transmembrane channel-like 4	−1,03	−2,04	
GPRC5A	G protein-coupled receptor, class C, group 5, member A	−1,02	−2,03	
TRHDE-AS1	TRHDE antisense RNA 1	−1,02	−2,03	
AQP5	aquaporin 5	−1,02	−2,03	
LINC00663	long intergenic non-protein coding RNA 663	−1,02	−2,02	
ZNF471	zinc finger protein 471	−1,01	−2,02	
CYP2J2	cytochrome P450, family 2, subfamily J, polypeptide 2	−1,01	−2,02	E
MEGF10	multiple EGF-like-domains 10	−1,01	−2,01	
FAXDC2	fatty acid hydroxylase domain containing 2	−1,00	−2,01	D
SLC13A2	solute carrier family 13 (sodium-dependent dicarboxylate transporter), member 2	−1,00	−2,01	

**Fig. 2:**
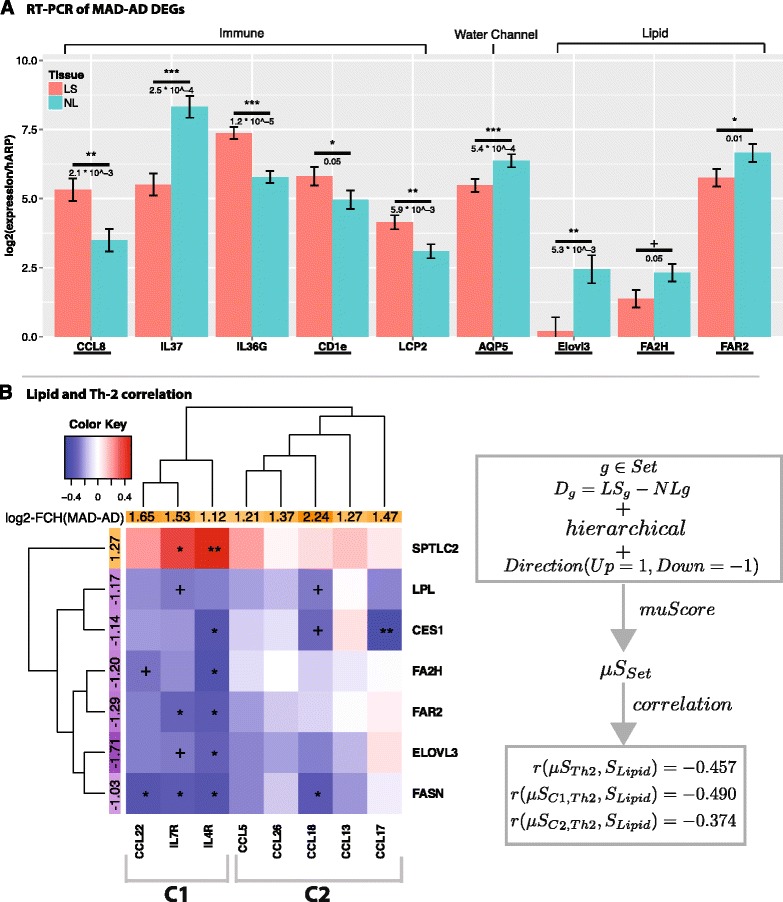
**a** Barplot representing means ± SEM of RT-PCR expression values for selected MADAD DEGs, normalized by *hARP* and log2 transformed. Underlined genes represent those not detected in any of the included datasets (IDD-genes). **b** Multivariate correlation between lipid and Th2-related genes dysregulations. Stars indicate the corresponding significance levels (+ ≤0.1, * ≤ 0.05, ** ≤0.01), while the correlations are shown from negative (−0.05, dark blue) to positive (0.05, dark red). The correlation data structure was assessed with Euclidean distance and complete agglomeration hierarchical clustering. Note that the right section gives a schematic overview of the gene set analysis and the corresponding correlation coefficients for the overall Th2 vs Lipid comparison and the sub-comparisons of C1 vs Lipid and C2 vs Lipid (see Additional file 15: Table E10 for corresponding BH adjusted p-values)

We further validated, using RT-PCR, several IDD-DEGs and top MADAD DEGs with plausible biologic relevance to AD, including immune (CCL8, CD1E, IL-37, IL-36G), structural (AQP5, a water channel) and lipid (FAR2, ELOVL3, FA2H) genes, which encode enzymes involved in fatty acid and ceramide metabolism (Fig. 2a). Immune-related genes (e.g. CCL8, LCP2, CD1E) and IL-36G, which was recently associated with psoriasis pathogenesis [70], were increased in AD LS skin. The only gene that showed increased expression in NL vs LS skin was IL-37, a negative immune regulator [71], consistent with past reports [72]. All structural and lipid metabolism genes showed downregulation in LS AD skin (P ≤ 0.05 for all), reinforcing the role of lipids and water channels in preserving barrier function in AD [73, 74].

### Suppression of lipid-related genes is coupled to increased Th2 activation

While we have previously shown inverse correlations between immune activation and terminal differentiation genes in AD lesions [11, 15], the relationship between immune activation and lipid metabolism genes has not been assessed in skin.

We thus investigated the relationship between expression of Th2-specific and epidermal lipid metabolism genes in the MADAD transcriptome. First we performed an unbiased database search for lipid related terms (see Methods) and chose genes with highest relevance scores. The correlation structure between lipid metabolism and immune genes in LS and NL skin is shown in Fig. 2b.

Unsupervised clustering identified two main sub-clusters in the Th2 gene-set and three main sub-clusters in the lipid metabolism gene-set. Cluster 3 consists only of SPTLC2, the only lipid metabolism gene with up-regulated expression in the MADAD transcriptome. This gene encodes serine palmitoyltransferase (SPT), a rate-limiting enzyme in sphingomyelin synthesis, whose elevated expression has been associated with increased barrier defects, including in AD [75–77].

Multivariate u-statistics correlate the dysregulation of immune and lipid metabolism genes [46]. Overall, an inverse correlation of −0.46 (*p* = 0.003) was obtained between Th2 and lipid subsets, supporting a proposed model of Th2 cytokine effects on lipid suppression [78]. Among Th2 genes, cluster 1 showed the highest negative correlation with lipid metabolism genes (−0.49; *p* = 0.001), and includes key AD genes (CCL22, IL-7R, and IL-4R) [63, 79–81]. Targeting IL-4R shows promise in early clinical trials as a possible therapeutic target for moderate-to-severe AD, and is now in phase 3 clinical trials for this disease (NCT02277743) [10, 18].

### Weighted gene co-expression network analysis

Weighted gene co-expression network analysis (WGCNA) offers insights into disease pathogenesis by studying weighted co-expression of genes within tissue samples [40]. This technique requires a large sample size [40, 82], which we were able to apply here for the first time in AD (see Methods).

**Fig. 3:**
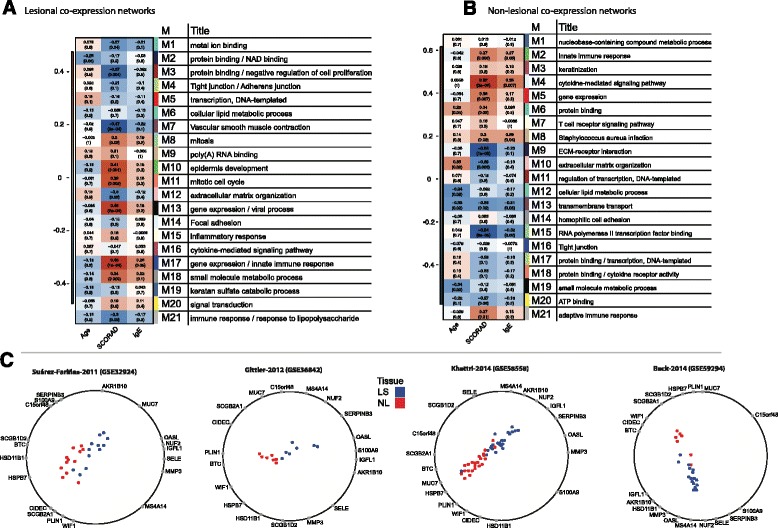
Module eigen-genes from weighted gene co-expression network analysis (WGCNA), correlated with Age, SCORAD and IgE, including annotation of top overrepresented BP GO-terms, KEGG Pathways or curated inflammatory skin diseases related gene-sets in (**a**) Lesional and (**b**) Non-lesional co-expression networks. Numbers in each cell represent the correlations with the respective trait and the associated p-values (see Additional file 16: Tables E11 and Additional file 17: Table E12 for corresponding BH adjusted p-values). **c** Radviz plots showing the separation of the lesional (LS) and non-lesional (NL) samples, as defined by the 19 genes signature identified by the MTGDR classifier (see Additional file 18: Table E13 for raw MTGDR results)

Using WGCNA, 21 distinct sub-networks were identified, and each was correlated with age, IgE level, and disease severity (measured by Scoring of Atopic Dermatitis/SCORAD) index (Fig. 3a-b, Additional file 12: Tables E7 and Additional file 13: Table E8). In LS skin, several networks showed significant positive correlations with SCORAD, including viral (M13) and innate immune response processes (M17)*,* emphasizing cutaneous immune reactions to viral and/bacterial pathogens in LS AD skin. Proliferation and epidermal processes were also correlated with SCORAD (M10, M11), with a trend for negative correlations between SCORAD and structural epidermal modules (M4, M12). In NL skin, immune-related networks such as cytokine receptor signaling pathway and innate immune response modules (M4, M2) [63, 83] were also positively correlated with SCORAD, while extracellular matrix (ECM) organization (M10) and ECM-receptor interaction (M9) modules were negatively correlated (Fig. 3b). Interestingly, a trend for a positive correlation was observed between SCORAD and *Staphylococcus aureus* (*S.aureus*) infection in NL skin (M8), with possible clinical relevance, since AD patients are often colonized with *S.aureus*, even in NL skin [4, 84]. Similar but weaker correlations were seen with IgE, while minimal correlations where found with age (Fig. 3a-b).

### A robust genomic AD classifier

Because clinical trials with specific and broad therapeutics are being tested in AD [5, 85], establishing a reliable gene set to discriminate between diseased and normal-appearing skin in AD is crucial. Here, we applied the classification algorithm Meta Threshold Gradient Directed Regularization (MTGDR) [41, 42], to determine the smallest set of genes that distinguishes LS from NL AD skin (see Methods). The final model identifies 19 genes (Fig. 3c and Table 2), including both epidermal and dermal genes, emphasizing the importance of both compartments to the disease model.

**Table 2:** MTGDR robust classifier genes, compared to treatment response, and indication of compartmental allocation as defined by Esaki et al. [63]

SYMBOL	LAYER	logFCH (MAD-AD)	logFCH (Dupilumab, 300 mg, W4)	logFCH (Cyclosporin, W2)	logFCH (Cyclosporin, W12)	logFCH (UVB, W12)	Genename	Recovery (Dupilumab, 300 mg, W4)	Recovery (Cyclosporin, W2)	Recovery (Cyclosporin, W12)	Recovery (UVB, W12)
IGFL1	E	3.5	−2.4^a^	−2.6^a^	−3.6^a^	−2.3^a^	IGF-like family member 1	68.60	74.81	101.60	64.33
OASL	D	2.9	−2.7^a^	−2.7^a^	−3.3^a^	−1.8^a^	2'-5'-oligoadenylate synthetase-like	93.07	93.24	112.20	60.33
SELE	D	2.4	−2.9^a^	−1.6^a^	−1.6^a^	−2.3^a^	selectin E	121.06	66.99	66.24	94.94
AKR1B10	E	3.2	−1.3	−3.2^a^	−4.0^a^	−3.0^a^	aldo-keto reductase family 1, member B10 (aldose reductase)	42.39	100.29	127.48	95.22
MS4A14	D	1.4	−2.0^a^	−1.2^a^	−1.4^a^	0.0	membrane-spanning 4-domains, subfamily A, member 14	149.43	84.71	102.42	0.00
NUF2	E	1.4	−2.2^a^	−1.4^a^	−1.4^a^	−0.9	NUF2, NDC80 kinetochore complex component	155.33	96.79	96.65	59.46
S100A9	E	3.8	−1.1	−3.7^a^	−4.5^a^	−2.2^a^	S100 calcium binding protein A9	27.95	96.75	116.47	57.26
SERPINB3	E	3.8	−1.9	−3.2^a^	−3.8^a^	−2.4^a^	serpin peptidase inhibitor, clade B (ovalbumin), member 3	50.29	85.47	101.40	62.98
C15orf48	E	1.0	−0.9	−1.0	−1.3^a^	−2.1^a^	chromosome 15 open reading frame 48	93.70	103.85	131.53	211.69
MMP3	D	1.1	−4.0^a^	−2.0^a^	−0.9	−0.3	matrix metallopeptidase 3 (stromelysin 1, progelatinase)	374.65	181.39	81.06	25.91
MUC7		−1.2	−0.1	0.0	0.0	1.1	mucin 7, secreted	−9.82	0.00	0.00	87.60
HSPB7	D	−1.6	1.6	0.6	1.7^a^	0.2	heat shock 27 kDa protein family, member 7 (cardiovascular)	102.98	37.95	107.88	13.51
SCGB1D2	D	−1.2	0.3	0.5	1.2^a^	0.0	secretoglobin, family 1D, member 2	25.96	43.28	100.97	0.00
BTC	E	−2.1	−0.1	1.0	1.3^a^	1.3^a^	betacellulin	−6.53	48.15	65.56	65.17
CIDEC		−1.7	2.1	1.2^a^	1.8^a^	0.0	cell death-inducing DFFA-like effector c	126.22	72.11	106.23	−2.38
HSD11B1	E	−2.3	−1.8	0.1	2.1^a^	1.0^a^	hydroxysteroid (11-beta) dehydrogenase 1	−77.60	5.69	91.93	44.65
PLIN1		−1.9	1.9	1.7^a^	2.0^a^	0.3	perilipin 1	98.89	85.20	103.82	12.83
SCGB2A1	D	−1.8	−0.1	0.9	1.7^a^	1.5^a^	secretoglobin, family 2A, member 1	−7.33	50.36	92.70	80.79
WIF1	D	−2.2	0.7	1.2	2.1^a^	1.3^a^	WNT inhibitory factor 1	29.58	52.56	92.11	58.40
E = Epidermis, D = Dermis (Esaki et al. [63])							Average	76.78	72.61	94.64	57.51

^a^Indicate FDR ≤ 0.05

To assess the translational validity of these 19 discriminating genes, we investigated the effect of various therapies on gene expression using previously published studies with Dupilumab (300 mg, 4 weeks of treatment) [10], Cyclosporin A (CsA; at 2 and 12 weeks) [12], and narrow band UVB/NB-UVB (12 weeks of treatment) [11], shown in Table 2. Reversal of disease phenotype to the NL state was observed in 18 of the 19 discriminating genes. Overall, a higher reversal was seen with CsA, a broad immune suppressant [86, 87], particularly after 12 weeks of treatment, with an average recovery of 94.64 % (see Table 2).

Among the genes down-regulated with treatment are inflammatory genes (S100A9, SELE) previously associated with AD [9, 59, 88]. Genes up-regulated with treatment (particularly with long-term CsA) include perilipin [89] and hydroxysteroid dehydrogenase [90], which are involved in lipid and steroid metabolism, respectively.

The LS and NL dysregulation in the MADAD correlates with the treatment change induced by CsA (*r* = 0.99; *p* = 2.5*10^−14^), dupilumab (*r* = 0.45; *p* = 0.189), and UBV (*r* = 0.90,*p* = 0.001) treatments, leading to restoration of gene expression similar to that seen in NL skin (Additional file 14: Figure E4).

## Discussion

A meta-analytic approach utilizes statistical processing and analysis to merge microarray studies from various populations and investigators, resulting in a single value that represents the estimated differential expression level of a gene between LS and NL skin. Combining multiple studies in a meta-analysis produces findings that more precisely reflect the differential expression of genes in a population, representing an accurate molecular characterization of a disease with increased power compared to individual analyses.

To address potential issues regarding meta-analysis application in gene expression studies, including laboratory effects, variations between probes and differential platforms, we planned this analysis beginning with data selection, through pre-processing and filtering, and finally to the meta-analysis model. This pipeline included the adjustment for study-related batch effects to minimize superficial inter-study discordance caused mainly by random noise and technical differences. Although in general this adjustment risk may confound true biological differences, we found no differences in disease severity, IgE and age across the four cohorts and thus feel confident that true biological differences are unlikely to be confounded. The resulting list of DEGs is presented here as a Meta-Analysis Derived Atopic Dermatitis (MADAD) transcriptome, utilizing 4 separate microarray studies for a total of 97 samples. It is our hope that the MADAD will prove helpful to investigators who may benefit from our robust characterization of the AD phenotype.

Similar to the MAD psoriasis transcriptome, our meta-analysis approach resulted in a more concise number of DEGs than that found in the individual studies [25]. However, this set of DEGs provides a more biologically relevant and powerful AD phenotype compared with previous studies [12], since important inflammatory and barrier pathways are more significantly enriched compared to individual studies, as is often observed with meta-analysis derived transcriptomes [91].

Our transcriptome is the first association of AD genomic fingerprinting with the atherosclerosis signaling pathway, which includes genes associated with vascular inflammation (SELE, IL-37, S100A8) [63, 77]. SELE has been independently associated with coronary heart disease and carotid artery atherosclerosis, and its expression in the vascular endothelium of the dermis of AD patients has also been observed [51, 88]. This supports the emerging view of AD as a systemic disease, which, like psoriasis, extends far beyond the skin [92, 93].

We have recently shown increased systemic immune activation in blood of moderate-to-severe AD patients among both skin homing and systemic T-cell subsets [94]. Furthermore, when comparing blood moderate-to-severe psoriasis and AD patients, we have shown that AD is associated with systemic activation and increased polar differentiation of effector and memory T-cell subsets, with higher and persistent activation within skin homing subsets. (Czarnowicki et al.-In press) AD patients also demonstrated higher levels of ICOS activation in circulating skin-homing subsets, (Czarnowicki- In press) consistent with the significant overrepresentation of ICOS signaling in our IPA analysis.

In large cohort studies, AD has also been recently associated with a variety of systemic diseases including inflammatory bowel disease [95, 96], Type 1 Diabetes Mellitus [97], and ADHD [98], providing further evidence for its systemic nature. Additionally, AD, like psoriasis, was recently shown to be associated with increased vascular inflammation using CT imaging [99–101]. The association of AD with systemic involvement emphasizes the great unmet need among severe AD patients for systemic therapeutic approaches, which are now in clinical trials (NCT00638989) [10, 18].

An interesting association between barrier defects and vascular inflammation in AD may be represented by the gene SPTLC2, which encodes a SPT, the rate-limiting enzyme in de novo synthesis of sphingomyelin and ceramides, and which has also been shown to influence atherosclerosis. Plasma sphingomyelin level was found to be an independent risk factor for coronary artery disease and is associated with subclinical atherosclerosis in humans [102, 103]. In ApoE knockout mice, inhibition of SPT resulted in improved lipid profiles and prevented the development of atherosclerotic lesions [104]. Sphingomyelin is proposed to affect atherosclerosis by influencing lipid metabolism and regulating cell proliferation and apoptosis to modulate plaque growth and stability [105]. SPTLC2 increases with epidermal barrier abnormalities [75], as was observed in our MADAD transcriptome, and may be related to the previously unacknowledged systemic vascular inflammation in AD.

Lipid and differentiation abnormalities represent the hallmark of defective barrier function in AD [106, 107]. The Th2 cytokine effects on inhibition of terminal differentiation genes (e.g. filaggrin, loricrin) have been well documented by in vitro [1, 78] and in vivo studies [15, 108]. While the effects of Th2 cytokines on suppression of epidermal lipids have become recently available in model systems [78], our paper is the first to show negative correlations between expression of Th2 cytokines and epidermal lipids. It is established that AD LS skin shows alterations in lipid composition of the stratum corneum [109], with decreases in long-chain ceramides and free fatty acids in addition to disorganized lipid structure [107]. While Th1 cytokines, including TNFα and IFNγ, have been shown to induce ceramide synthesis, Th2 cytokines (including IL-4) inhibit the production of ceramides necessary for proper barrier function [76, 110]. Our findings support these in vitro models, and show significant negative correlations between increased Th2 cytokine production and decreased lipid expression.

We also identified for the first time as differentially expressed in AD several genes involved in lipid metabolism. These include FA2H, encoding protein essential to the de novo synthesis of specific ceramides that are critical in maintaining the permeability barrier of the epidermis [111, 112], and ELOVL3, encoding a protein involved in the elongation of long chain fatty acids and essential in prevention of transepidermal water loss [113, 114]. While not involved in fatty acid synthesis, aquaporin 5/AQP5 is a water channel that was found to be decreased in LS AD skin [115–117].

The network analysis of MADAD DEGs shows a significant correlation between AD disease activity/SCORAD and the *S. aureus* module in NL skin, emphasizing the role of colonization and infection in the onset of immune activation in background skin. *S. aureus* colonization/infection has been shown to occur at significantly higher rates in both LS and NL AD skin compared to healthy controls [118–120], and was shown to induce Th2 and Th22 immune activation [84, 121–123]. In LS skin, viral and innate immune process modules were significantly positively correlated with SCORAD, emphasizing the association between reactions to external pathogens and active inflammation in AD [121].

The treatment effect on the 19 discriminatory genes highlights the diverse mechanistic effects of these therapies with several observations worth mentioning. First, MMP3, a marker of general inflammation, displays an impressive recovery with the targeted therapy dupilumab compared to the more nonspecific immune suppressant treatments. This inflammatory gene improved over 300 % in only 4 weeks of treatment with dupilumab, while long-term treatment with CsA and UVB did not reach 100 % recovery. This may suggest the ability of dupilumab to suppress immune dysregulation in AD in a shorter timeframe than with less specific agents. Another noteworthy gene is Selectin E, encoding a protein involved in leukocyte extravasation, which also showed higher levels of recovery in dupilumab compared to CsA and UVB therapy. The difference in these markers may be related to the mechanism of action of each drug; UVB has direct effects on keratinocytes and thus mainly mediates signals originating there, while CsA is a nonspecific inhibitor of T-cells, B-cells and related pathways. Dupilumab more specifically modulates IL-4/IL-13 signaling, which has been implicated in the pathogenesis of AD, and this may account for the impressive recovery seen in genes central to the inflammatory response generated by AD.

## Conclusions

This meta-analysis provides a stable and robust AD transcriptome worthy of future biomarker selection and evaluation of treatment response. The value of a standardized AD transcriptome will only increase with the bench-to-bedside translational approach, as a standardized measure of treatment response that cannot be confounded with placebo effects. This meta-analysis provides an integrative model of AD, emphasizing both immune and barrier abnormalities and also highlighting the systemic nature of the disease.

## Additional files

Additional file 1: **Supplementary materials and methods.** (DOCX 111 kb)

Additional file 2: Table E9. Primer IDs for RT-PCR analysis. (XLSX 37 kb)

Additional file 3: Figure E1. A) Prisma. B) MADAD Datasets. C) Meta-Analysis Workflow. (PDF 608 kb)

Additional file 4: Table E1. All datasets intially considered before applying inclusion/exclusion criteria. (DOCX 65 kb)

Additional file 5: Table E2. Consensus of re-analyized included datasets (by Probes). (XLSX 60 kb)

Additional file 6: Figure E2. QQ plot. Comparison of parameters. (PDF 1005 kb)

Additional file 7: Table E3. ALL MADAD DEGs (FCH ≥ 2, FDR ≤ 0.05). (XLSX 171 kb)

Additional file 8: Table E4. MADAD – Ingenuity Pathway Analysis. (XLSX 33 kb)

Additional file 9: Table E5. Khattri 2014 (GSE58558) – Ingenuity Pathway Analysis. (XLSX 26 kb)

Additional file 10: Figure E3. A) Inflammatory Skin Disease Gene Sets. B) MADAD IDD-DEGs IPA Canonical Pathways. (PDF 85 kb)

Additional file 11: Table E6. IPA Pathways and GO terms IDD genes. (XLSX 9 kb)

Additional file 12: Table E7. Overrepresented Hub30 Genes and module names. (LS) (XLSX 38 kb)

Additional file 13: Table E8. Overrepresented Hub30 Genes and module names. (NL) (XLSX 47 kb)

Additional file 14: Figure E4. logFCHs correlations of MADAD and treatments A) Dupilumab (300mg), B) Cyclosporin (W12), and C) UVB. (PDF 42 kb)

Additional file 15: Table E10. Adjusted P-values for the significance indicators in Figure 2B. (XLSX 8 kb)

Additional file 16: Table E11. BH adjusted p-values WGCNA LS Modules. (XLSX 9 kb)

Additional file 17: Table E12. BH adjusted p-values WGCNA NL Modules. (XLSX 9 kb)

Additional file 18: Table E13. MTGDR results. (XLSX 9 kb)

## Abbreviations

AD: Atopic dermatitis
CsA: Cyclosporin A
DEGs: Differentially expressed genes (logFCH ≥ 1 and fdr ≤ 0.05)
FDR: False Discovery Rate
FEM: Fixed-effects model (special case of REM)
ICC: Integrated Correlation Coefficient
IDD: Integration-driven discovery
IgE: Immunoglobulin E
IPA: Ingenuity pathway analysis
LS: Lesional
MAD-AD: Meta-analysis derived AD
MTGDR: Meta Threshold Gradient Directed Regularization
NL: Non-lesional
PRISMA: Preferred Reporting Items for Systematic Reviews and Meta-Analyses
REM: Random-effects model
RT-PCR: Quantitative Reverse Transcription PCR
RPLP0: Ribosomal protein, large, P0 (formerly termed *human acidic ribosomal protein P0*/hARP)
S. aureus: Staphylococcus aureus
SCORAD: Scoring of Atopic Dermatitis
SPT: Serine palmitoyltransferase
WGCNA: Weighted gene co-expression network analysis
