# Supplementary material for: Meta-analysis derived atopic dermatitis (MADAD) transcriptome defines a robust AD signature highlighting the involvement of atherosclerosis and lipid metabolism pathways
Source: BMC Med Genomics. 2015 Oct 12;8:60. doi: 10.1186/s12920-015-0133-x (PMC4603338; doi:10.1186/s12920-015-0133-x)
Supplement: Supplementary file 1 — Supplementary materials and methods. (DOCX 111 kb) [file 12920_2015_133_MOESM1_ESM.docx]

**SUPPLEMENTARY MATERIALS AND METHODS**

**Sample Collection**

All samples were collected according to the Preferred Reporting Items for Systematic Reviews and Meta-Analyses (PRISMA) statement.

A total of 28 datasets were detected in Gene Expression Omnibus (GEO) but only datasets evaluating expression data of LS vs. NL skin samples of AD patients were retained for analyses. Datasets run on platforms other than the HGU133Plus 2 chip, subject to treatments, or with non-randomly selected NL or LS samples (e.g. FLG homozygous/heterozygous loss of function mutation), and datasets without NL samples were excluded. When overlapping samples were found between datasets, only one copy was kept.

In total, four microarray datasets were included (GSE32924, GSE36842, GSE58558, GSE59294), including 97 samples (54 LS and 43 NL). “Minimum Information about a Microarray Experiment” (MIAME) was available for all studies.

**Pre-processing and Expression Analysis**

The pre-processing and statistical analysis of the raw microarray data was carried out using *R* and *Bioconductor* packages.

The raw expression data of the four included datasets were combined, summarized, and normalized using the *GCRMA* algorithm with *quantile-normalization,* taking into account the individual probes’ affinities towards non-specific binding by utilizing probe specific sequence information.

Possible batch effects between the four chosen datasets were accessed by *principal component analysis* (*PCA*) and adjusted for by the empirical bayes method *ComBat* from the *sva* package.

This was followed by a two-level filtering procedure, where the combined datasets were filtered first by the per probe-set *Standard Deviation* (*SD*>0.1) and second by the *Integrated Correlation Coefficient* (*ICC*; mean ICC>0.2) The final analysis was conducted on the remaining 21082 probe sets (out of 54675 total probe sets). The before and after filtering correlations between the datasets was accessed by the average pairwise maximum canonical correlations, calculated by the *MergeMaid* package.

To assess the individual studies effects, model estimation and hypothesis testing for the LS versus NL comparison was performed using the mixed-effect framework of the *limma* package, with patients as a random effect and tissue as a fixed effect. P-values were adjusted for multiple testing using the *Benjamini-Hochberg procedure,* with criteria for differentially expressed genes (DEGs) of absolute fold change (|FCH|) ≥ 2.0 and a false discovery rate (FDR) ≤ 0.05.

**Meta-analysis**

The formal *random effects model (REM)*, as described by Choi and colleagues in 2003, is given below. Let µ*_i_* be the unbiased effect mean effect size for gene *i* (*i*=1,…,*I*), and *Y_ij_* the measured effect size for gene *i* study *j* (*j*=1,…,*J*). Then for gene *i* and dataset/study *j*:

$$Y_{ij}= \theta_{ij}+\varepsilon_{ij} , \varepsilon_{ij} \sim N\left( 0, s_{ij}^{2} \right)$$

$$\theta_{ij}= \mu_{i}+\delta_{ij} , \delta_{ij} \sim N(0, \tau_{i}^{2} )$$

,where $\tau_{i}^{2}$ describes the cross-study variance for gene *i*. An estimate of this variance term is readily found by the DerSimonian and Laird (DL) moments estimator. The error term of the observed effect size is characterized by the within-study variance $s_{ij}^{2}$ for a given gene *i* in a given study *j*. Both the measured effect size $Y_{ij}$ and the within-study variance are readily assessable from the initial individual analysis of the included datasets. For gene *i,* the parameter of interest $\mu_{i}$, the average effect size, and its standard error (*se*) are estimated making use of the DL point estimate for $\tau_{i}^{2}$:

$$\hat{\mu}_{i}\left( \tau_{i}^{2} \right)= \frac{\sum_{j=1}^{J} {{(s}_{ij}^{2}+\tau_{i}^{2})}^{-1} y_{ij}}{\sum_{j=1}^{J} {{(s}_{ij}^{2}+\tau_{i}^{2})}^{-1}} , se\left[ \hat{\mu}_{i}\left( \tau_{i}^{2} \right) \right]= \sqrt{\frac{1}{\sum_{j=1}^{J} {{(s}_{ij}^{2}+\tau_{i}^{2})}^{-1}}}$$

The *fixed effect model (FEM)* is a special case of the *REM*, assuming the observed differences in effect sizes to be from sampling error alone, i.e. $\tau_{i}^{2}=0$ .

Both a *FEM* and a *REM* were applied in order to effectively estimate the true effect size for each probe. Quantile-quantile plots of Cochran’s Q sample quantiles versus the theoretical quantiles of the $\chi_{3}^{2}$-distribution (from the general $\chi_{k-1}^{2}$-distribution, with *k* number of datasets = 4) were applied to assess the most appropriate meta-analysis model (Fig E3).

These estimation and calculation steps were carried out making use of the package *GeneMeta.*

**Post-processing**

The MAD-AD transcriptome was subject to multiple downstream analyses methods. Integration-driven discovery (IDD) DEGs were defined as those not described in any of the included studies. To explore the functional annotations, overrepresentation analysis was carried out for BP GO-terms and KEGG pathways (both in DAVID), Ingenuity Pathways (*IPA –* www.ingenuity.com , as described), and on previously reported gene-sets.

Furthermore, the normalized LS and NL expression data were subject to Weighted Gene Co-Expression Networks Analysis (WGCNA), in order to detect clusters (modules) of correlated genes and their respective hub genes. The modules of this analysis were subject to trait correlation and corresponding gene-set overrepresentation analysis (see Tables E11-12 for corresponding BH adjusted p-values).

Meta Threshold Gradient Directed Regularization (MTGDR) method proposed by Ma and Huang (2009) was used to select the same set of disease-associated genes while allowing for varied estimates of those genes across different experiments, as we have previously published (see Table E13 for raw MTGDR results).

RT-PCR was analyzed as previously described [43] using the primers and probes listed in Table E9. The expression data from these experiments were analyzed in R applying a *mixed effect model* with tissue as fixed effect and patients as random effect.

Lipid genes were defined as genes related to one of the four groups: *Ceramides*, *Free Fatty Acids*, *Sphingolipids*, and *Cholesteryl Esters*. We searched the genecards database for those terms, and included all genes with a relevance score ≥ 10. Pairwise Pearson correlations were calculated between the gene and patient specific deregulations. Multivariate correlations between sets of genes were calculated making use of the gene set specific µ-scores calculated by the *muStat* package (see Table E10 for BH adjusted Pvalues for Figure 2B).
