# Supplementary material for: Meta-analysis derived atopic dermatitis (MADAD) transcriptome defines a robust AD signature highlighting the involvement of atherosclerosis and lipid metabolism pathways
Source: BMC Med Genomics. 2015 Oct 12;8:60. doi: 10.1186/s12920-015-0133-x (PMC4603338; doi:10.1186/s12920-015-0133-x)

Figure E1

A)

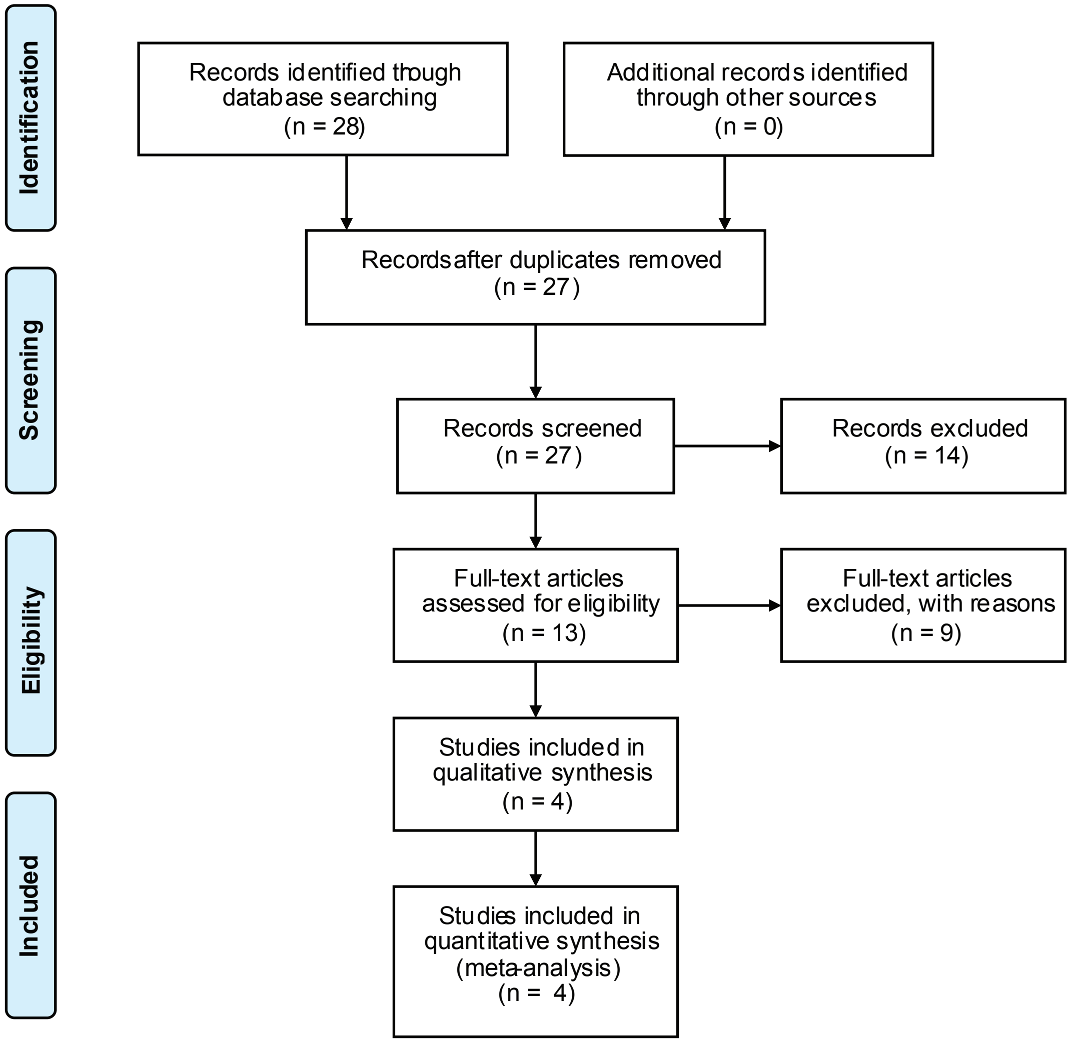

B) MAD-AD Datasets

Author	Database ID	Patients	Paired (LS/NL)	ChipID
Beck et al. 2014	GSE59294	16	7	GPL570
Khattri et al. 2014	GSE58558	19	16	GPL570
Gittler et al. 2012	GSE36842	7	7	GPL570
Suárez-Fariñas et al. 2011	GSE32924	14	11	GPL570
Total		56	41	

C) Meta-Analysis Workflow

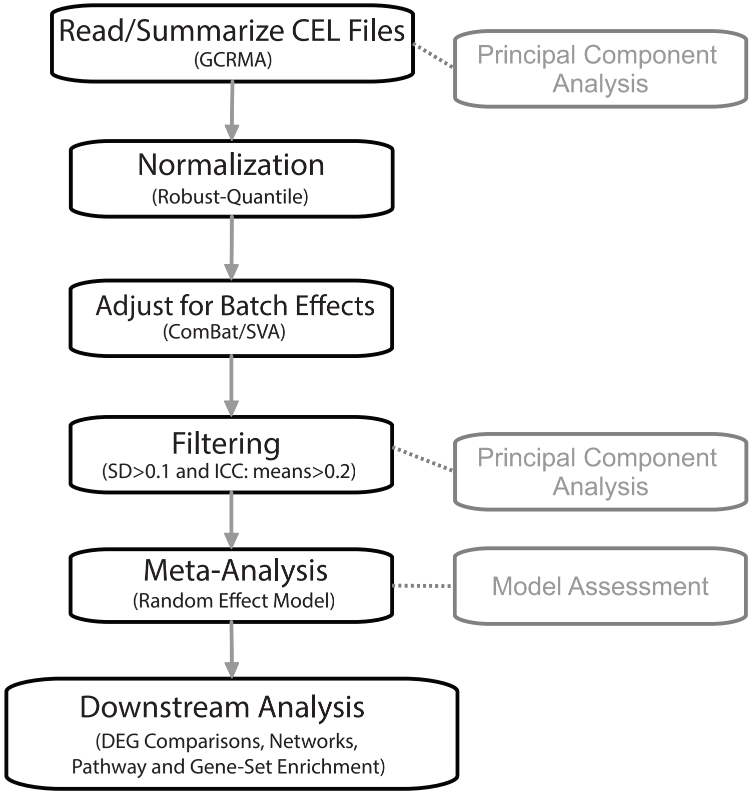

Supplement: Supplementary file 3 — A) Prisma. B) MADAD Datasets. C) Meta-Analysis Workflow. (PDF 608 kb) [file 12920_2015_133_MOESM3_ESM.pdf]
