# Supplementary material for: Meta-analysis derived atopic dermatitis (MADAD) transcriptome defines a robust AD signature highlighting the involvement of atherosclerosis and lipid metabolism pathways
Source: BMC Med Genomics. 2015 Oct 12;8:60. doi: 10.1186/s12920-015-0133-x (PMC4603338; doi:10.1186/s12920-015-0133-x)
Supplement: Supplementary file 4 — All datasets intially considered before applying inclusion/exclusion criteria. (DOCX 65 kb) [file 12920_2015_133_MOESM4_ESM.docx]

| **Author** | **Year** | **GSE/eMTAB** | **Sample size** | **LS** | **NL** | **NN** | **Status** | **Comment** | **ChipID** |
| --- | --- | --- | --- | --- | --- | --- | --- | --- | --- |
| Beck | 2014 | [GSE59294](http://www.ncbi.nlm.nih.gov/geo/query/acc.cgi?acc=GSE59294) | 23 | 16 | 7 | 0 | **IN** |  | [GPL570](http://www.ncbi.nlm.nih.gov/geo/query/acc.cgi?acc=GPL570) |
| Khattri | 2014 | [GSE58558](http://www.ncbi.nlm.nih.gov/geo/query/acc.cgi?acc=GSE58558) | 35 | 18 | 17 | 0 | **IN** |  | [GPL570](http://www.ncbi.nlm.nih.gov/geo/query/acc.cgi?acc=GPL570) |
| Gittler | 2012 | [GSE36842](http://www.ncbi.nlm.nih.gov/geo/query/acc.cgi?acc=GSE36842) | 29 | 7 | 7 | 15 | **IN** |  | [GPL570](http://www.ncbi.nlm.nih.gov/geo/query/acc.cgi?acc=GPL570) |
| Suárez-Fariñas | 2011 | [GSE32924](http://www.ncbi.nlm.nih.gov/geo/query/acc.cgi?acc=GSE32924) | 33 | 13 | 12 | 8 | **IN** |  | [GPL570](http://www.ncbi.nlm.nih.gov/geo/query/acc.cgi?acc=GPL570) |
| Han | 2012 | [GSE35582](http://www.ncbi.nlm.nih.gov/geo/query/acc.cgi?acc=GSE35582) | 3 | 2 | 0 | 1 | **OUT** | No NL | [GPL9052](http://www.ncbi.nlm.nih.gov/geo/query/acc.cgi?acc=GPL9052) |
| Rebane | 2012 | [E-MTAB-728](http://www.ebi.ac.uk/arrayexpress/experiments/E-MTAB-728/) | 6 | 3 | 0 | 3 | **OUT** | No NL | [A-MEXP-1171](http://www.ebi.ac.uk/arrayexpress/arrays/A-MEXP-1171/?ref=E-MTAB-728) |
| De Benedetto | 2011 | [GSE26952](http://www.ncbi.nlm.nih.gov/geo/query/acc.cgi?acc=GSE26952) | 12 | 5 | 0 | 7 | **OUT** | No NL | [GPL2700](http://www.ncbi.nlm.nih.gov/geo/query/acc.cgi?acc=GPL2700) |
| Winge | 2011 | [E-MTAB-768](http://www.ebi.ac.uk/arrayexpress/experiments/E-MTAB-768/) | 20 | 15 | 0 | 5 | **OUT** | No NL | A-AFFY-141 |
| Guttman-Yassky | 2009 | [GSE16161](http://www.ncbi.nlm.nih.gov/geo/query/acc.cgi?acc=GSE16161) | 18 | 9 | 0 | 9 | **OUT** | No NL | [GPL570](http://www.ncbi.nlm.nih.gov/geo/query/acc.cgi?acc=GPL570) |
| Olsson | 2006 | [GSE6012](http://www.ncbi.nlm.nih.gov/geo/query/acc.cgi?acc=GSE6012) | 20 | 10 | 0 | 10 | **OUT** | No NL | [GPL96](http://www.ncbi.nlm.nih.gov/geo/query/acc.cgi?acc=GPL96) |
| Rebane | 2012 | [E-MTAB-729](http://www.ebi.ac.uk/arrayexpress/experiments/E-MTAB-729/) | 10 | 3 | 3 | 4 | **OUT** | Chip Type and Small sample size |  |
| Plager | 2010 | [GSE5667](http://www.ncbi.nlm.nih.gov/geo/query/acc.cgi?acc=GSE5667) | 17 | 6 | 6 | 5 | **OUT** | Chip Type (Significant less genes) | GPL96, GPL97 |
| Tintle | 2011 | [GSE27887](http://www.ncbi.nlm.nih.gov/geo/query/acc.cgi?acc=GSE27887) | 17 | 9 | 8 | 0 | **OUT** | Duplicate data also used in GSE32924 | [GPL570](http://www.ncbi.nlm.nih.gov/geo/query/acc.cgi?acc=GPL570) |
| Rodríguez | 2014 | [GSE60709](http://www.ncbi.nlm.nih.gov/geo/query/acc.cgi?acc=GSE60709) | 33 | 12 | 7 | 14 | **OUT** | Design - Epidermal Shave | [GPL6947](http://www.ncbi.nlm.nih.gov/geo/query/acc.cgi?acc=GPL6947) |
| Jensen | 2012 | [GSE32473](http://www.ncbi.nlm.nih.gov/geo/query/acc.cgi?acc=GSE32473) | 30 | 30 |  |  | **OUT** | Design - Only lesionals, and two thirds of them treatet |  |
| de Jongh | 2005 | [GSE6601](http://www.ncbi.nlm.nih.gov/geo/query/acc.cgi?acc=GSE6601) | 6 | 6 | 0 | 0 | **OUT** | Design - PSO and AD only LS |  |
| Liang | 2013 | [E-MTAB-1428](http://www.ebi.ac.uk/arrayexpress/experiments/E-MTAB-1428/) | 543 |  |  |  | **OUT** | Design - cell cultures (expression in vitro) |  |
| Yoshikawa | 2013 | [GSE13709](http://www.ncbi.nlm.nih.gov/geo/query/acc.cgi?acc=GSE13709) | 48 |  |  |  | **OUT** | Design - cell cultures, BDK vs. NHEK |  |
| Billmann-Born | 2011 | [GSE22611](http://www.ncbi.nlm.nih.gov/geo/query/acc.cgi?acc=GSE22611) | 27 |  |  |  | **OUT** | Design - cell cultures (Flp-In HEK cells) treatet |  |
| Gutowska-Owsiak | 2012 | [GSE27533](http://www.ncbi.nlm.nih.gov/geo/query/acc.cgi?acc=GSE27533) | 6 |  |  |  | **OUT** | Design - cell culture (HaCaT cells) treatet |  |
| Hirakawa | 2011 | [GSE20706](http://www.ncbi.nlm.nih.gov/geo/query/acc.cgi?acc=GSE20706) | 9 | 6 | 3 | 0 | **OUT** | Design - cell based epidermal keratinocytes (NHEK), treated | [GPL6480](http://www.ncbi.nlm.nih.gov/geo/query/acc.cgi?acc=GPL6480) |
| Chisaguano | 2013 | [GSE48310](http://www.ncbi.nlm.nih.gov/geo/query/acc.cgi?acc=GSE48310) | 124 | 20 |  | 104 | **OUT** | Design - cell based and qRT-PCR, No NL | [GPL17344](http://www.ncbi.nlm.nih.gov/geo/query/acc.cgi?acc=GPL17344) |
| Cohen | 2012 | [GSE38039](http://www.ebi.ac.uk/arrayexpress/experiments/E-GEOD-38039/?query=Atopic%20Dermatitis&sortby=accession&sortorder=descending) | 6 |  |  |  | **OUT** | Design - cell based (HaCaT keratinocytes) |  |
| Titova | 2013 | [GSE48586](http://www.ncbi.nlm.nih.gov/geo/query/acc.cgi?acc=GSE48586) | 12 |  |  |  | **OUT** | Design - treated artificial human skin tissues |  |
| Quaranta | 2014 | [GSE57225](http://www.ncbi.nlm.nih.gov/geo/query/acc.cgi?acc=GSE57225) | 62 | 23 | 17 |  | **OUT** | Design - Co-affected patients (PSO+AD) | [GPL14550](http://www.ncbi.nlm.nih.gov/geo/query/acc.cgi?acc=GPL14550) |
| Dhingra | 2014 | [GSE60028](http://www.ncbi.nlm.nih.gov/geo/query/acc.cgi?acc=GSE60028) | 47 |  |  |  | **OUT** | Design - Contact dermatitis | [GPL570](http://www.ncbi.nlm.nih.gov/geo/query/acc.cgi?acc=GPL570) |
| Wong | 2012 | [GSE32245](http://www.ncbi.nlm.nih.gov/geo/query/acc.cgi?acc=GSE32245) | 54 |  |  |  | **OUT** | Design - dermatomyositis |  |
| Rettew | 2013 | [E-MTAB-62](http://www.ebi.ac.uk/arrayexpress/experiments/E-MTAB-62/) | 5372 |  |  |  | **OUT** | Design - combined publicly available data of mouse and humans | |
