# Supplementary figures and images for: Meta-analysis derived atopic dermatitis (MADAD) transcriptome defines a robust AD signature highlighting the involvement of atherosclerosis and lipid metabolism pathways
Source: BMC Med Genomics. 2015 Oct 12;8:60. doi: 10.1186/s12920-015-0133-x (PMC4603338; doi:10.1186/s12920-015-0133-x)

Quatiles of samples ( $Q=4.98$  ;  $\sigma^2=16.59$ )

## QQ plot

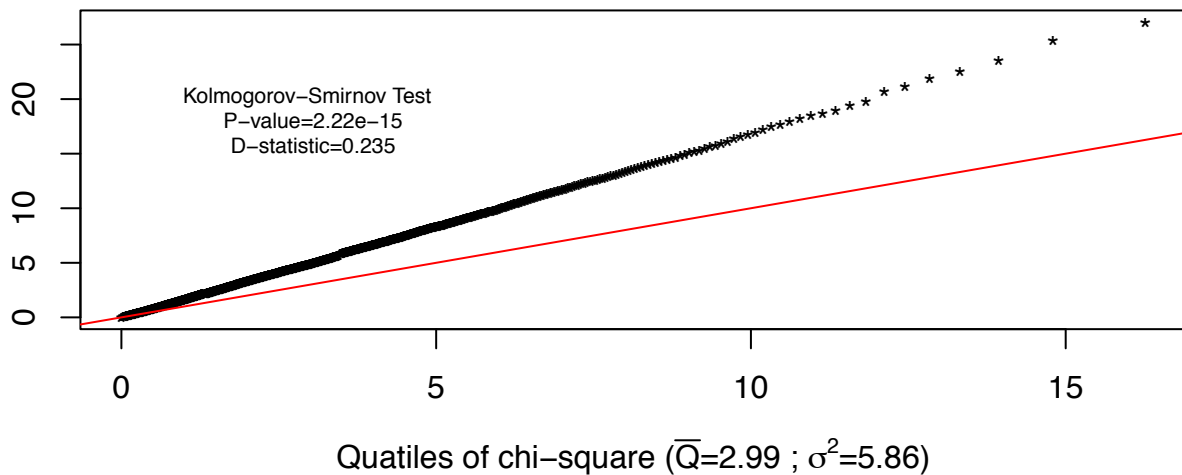

## Comparison of parameters

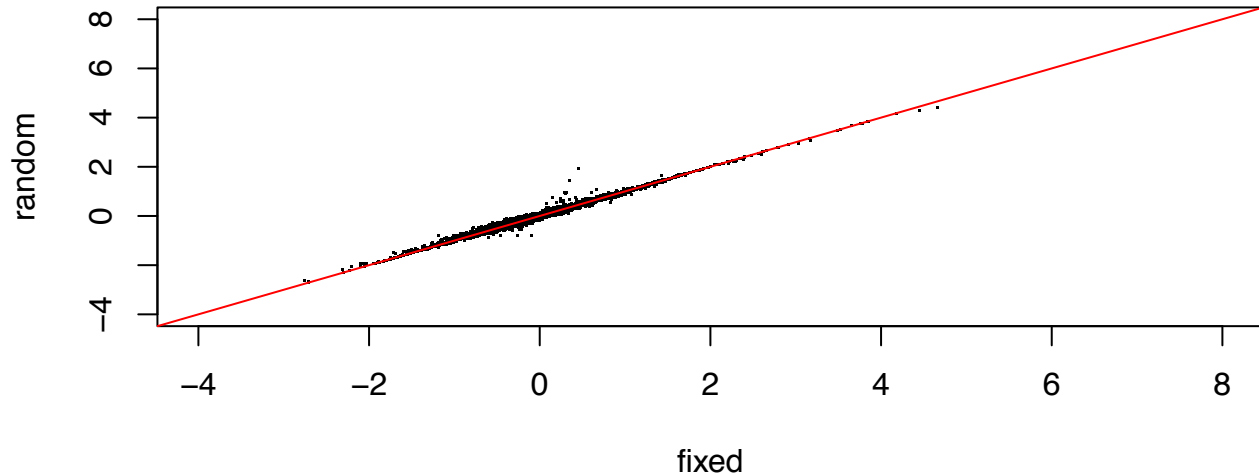

Supplement: Supplementary file 6 — QQ plot. Comparison of parameters. (PDF 1005 kb) [file 12920_2015_133_MOESM6_ESM.pdf]

Figure E3

A) Inflammatory Skin Disease Gene-Sets

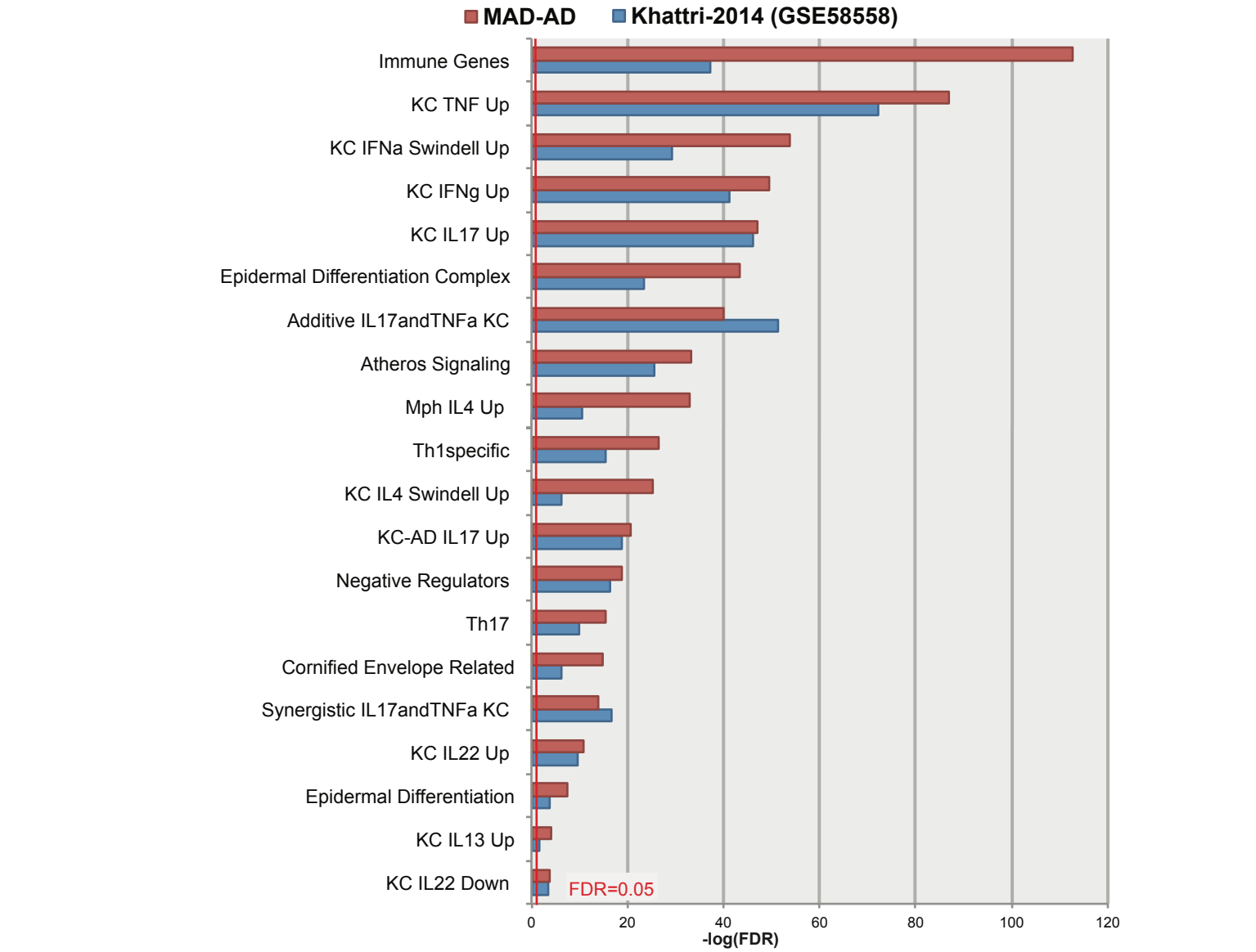

B) MAD-AD IDD-DEGs IPA Canonical Pathways

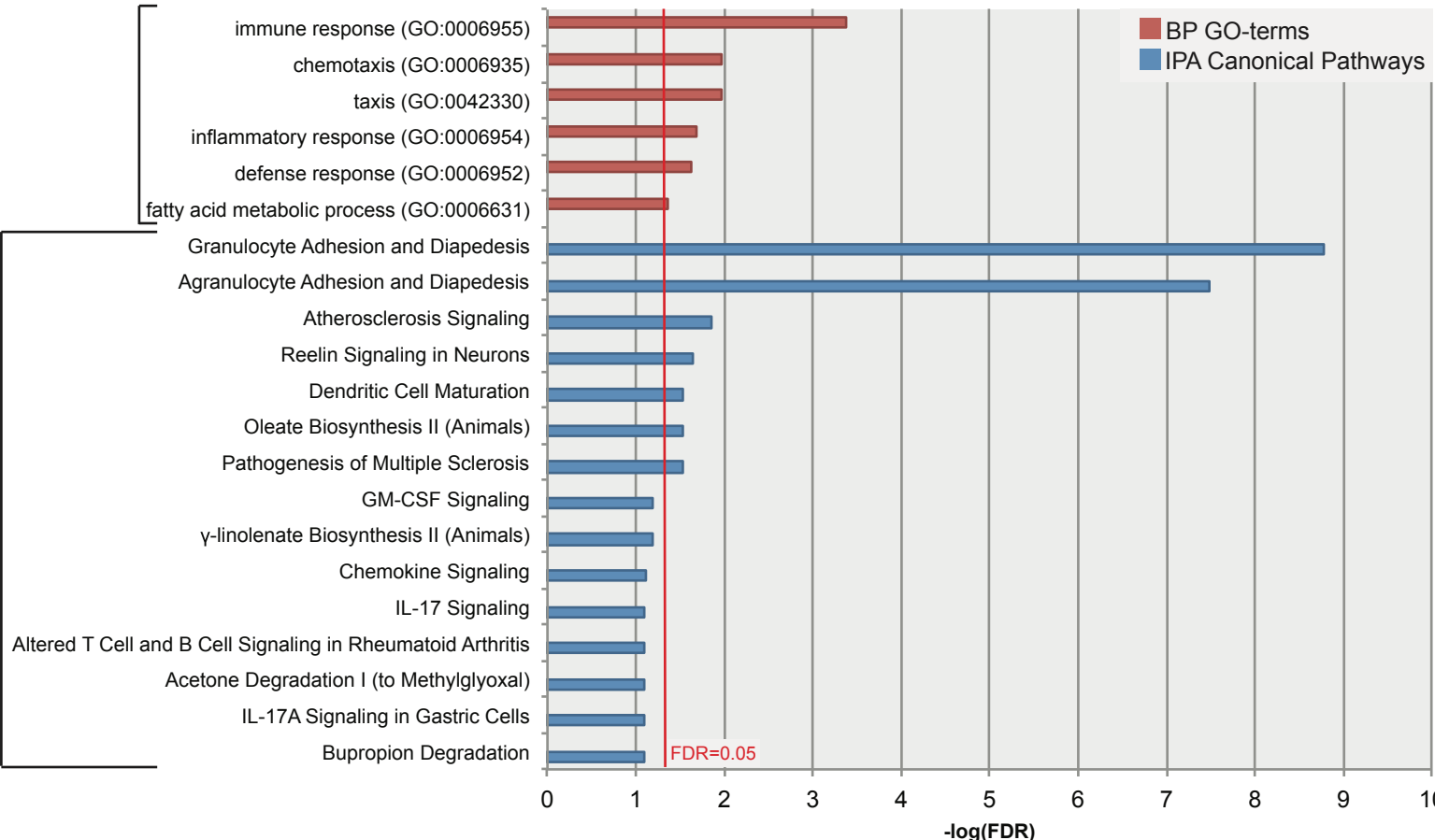

Supplement: Supplementary file 10 — A) Inflammatory Skin Disease Gene Sets. B) MADAD IDD-DEGs IPA Canonical Pathways. (PDF 85 kb) [file 12920_2015_133_MOESM10_ESM.pdf]

A)

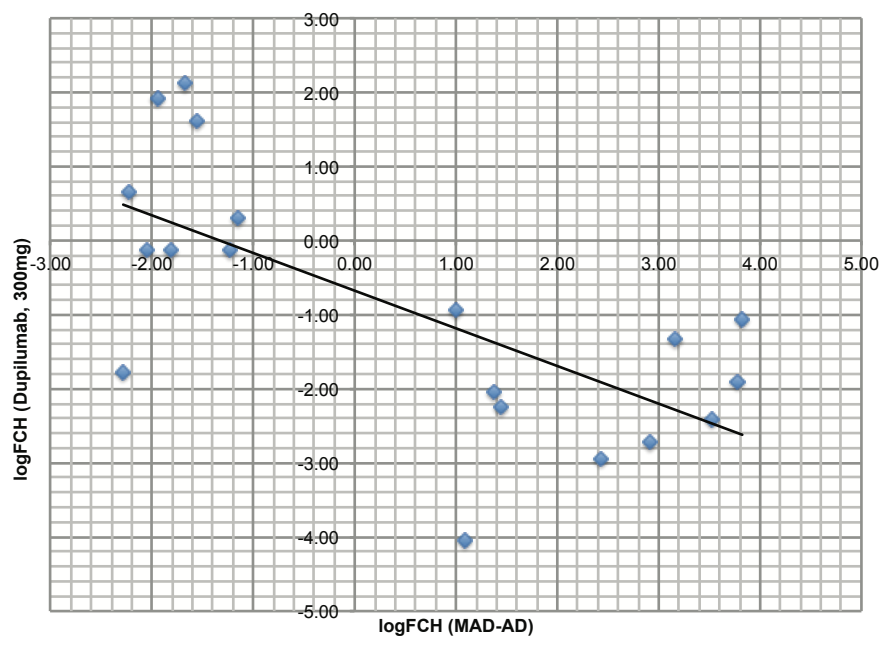

B)

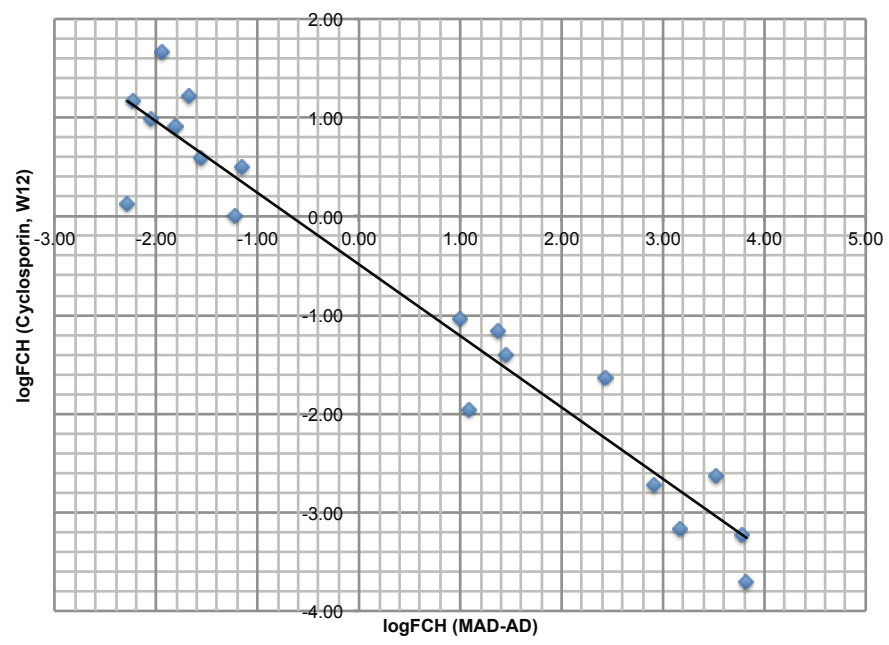

C)

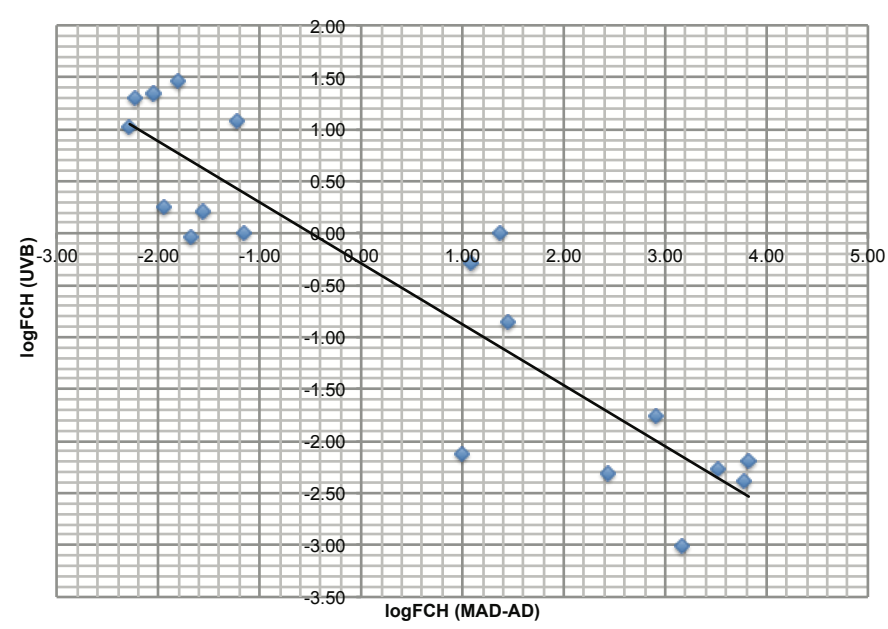

Supplement: Supplementary file 14 — logFCHs correlations of MADAD and treatments A) Dupilumab (300mg), B) Cyclosporin (W12), and C) UVB. (PDF 42 kb) [file 12920_2015_133_MOESM14_ESM.pdf]
